# Prevalence of Respiratory Polyomaviruses Among Pediatric Patients With Respiratory Symptoms in Singapore

**DOI:** 10.3389/fped.2018.00228

**Published:** 2018-08-17

**Authors:** Christophe Hansen-Estruch, Kristen K. Coleman, Koh C. Thoon, Jenny G. Low, Benjamin D. Anderson, Gregory C. Gray

**Affiliations:** ^1^Program in Emerging Infectious Diseases, Duke-NUS Medical School, Singapore, Singapore; ^2^Duke University School of Medicine, Durham, NC, United States; ^3^Department of Paediatrics, Infectious Disease Service, KK Women's and Children's Hospital, Singapore, Singapore; ^4^Department of Infectious Diseases, Singapore General Hospital, Singapore, Singapore; ^5^Division of Infectious Diseases, Global Health Institute and Nicholas School of the Environment, Duke University, Durham, NC, United States

**Keywords:** polyomavirus, WU, KI, respiratory illness, respiratory virus

## Abstract

**Background:** Although WU polyomavirus (WU) and KI polyomavirus (KI) have been demonstrated to infect the human respiratory tract, it remains unclear if WU or KI cause human disease. We sought to further investigate the relationship between WU and KI infection and respiratory disease in a pediatric population with respiratory symptoms in Singapore.

**Methods:** We conducted a cross-sectional study of pediatric patients with respiratory symptoms in a Singaporean pediatrics hospital. Upon consent, residual respiratory samples from pediatric inpatients, previously screened for common respiratory viruses, were collected and further screened for WU and KI using qPCR. The amplicons of positive samples were sequenced for confirmation. The severity of a patient's illness was assessed by chart review post-discharge looking for clinical markers of respiratory status such as presenting symptoms, diagnoses, and interventions.

**Results:** From December 2016 to April 2017, 201 patients with residual respiratory samples were enrolled in the study. The average age of all participants recruited was 45 months. WU and KI were detected in 13% (26/201) and 3% (6/201) of patients, respectively. Conducting bivariate and multivariate modeling, patients with WU or KI positivity were not at increased risk of SARI, need for additional oxygen, intravenous fluids, and did not receive additional oral antibiotics or bronchodilators during admission. In contrast, patients with RSV detections were at increased risk of requiring supplemental oxygen during hospital admission.

**Conclusion:** While limited in sample size, our pilot study data do not support the hypothesis that molecular evidence of WU or KI was associated with increased morbidity among a sample of general, pediatric patients with respiratory illness in Singapore.

## Introduction

Human polyomaviruses are non-enveloped DNA viruses known to cause disease in immunosuppressed human populations by infecting the urinary tract and central nervous system. However, recent clinical studies using molecular assays to monitor for polyomavirus infections in pediatric patients have revealed an association between respiratory disease and two newly discovered polyomaviruses, WU polyomavirus (WU), and KI polyomavirus (KI) (1–6). Recently, the International Committee on Taxonomy of Viruses has classified WU and Kl as polyomavirus species 4 and 3, respectively. Potential for these respiratory polyomaviruses to cause or worsen disease has been suggested by an observed positive relationship between real-time PCR Ct values from KI-positive patient samples and the severity of respiratory symptoms (7).

Even though WU and KI were only recently discovered in 2007, researchers have established a worldwide presence spanning North America, Europe, Asia (2, 8–13). The majority of respiratory polyomavirus studies have focused upon archived clinical samples and have not provided detailed clinical data regarding the symptoms experienced by polyomavirus infected patients (2, 8–13). As a result, the clinical implications and burden on health systems of WU and KI infection are not well known. Although WU and KI have been demonstrated to infect the human respiratory tract, it remains unclear how often WU and KI infections cause clinical signs or symptoms (14, 15). In this study, we sought to further investigate the relationship between respiratory disease and WU and KI infection by conducting surveillance for these viruses among Singaporean pediatric patients experiencing respiratory illness to evaluate the severity of their disease using clinical markers of respiratory status or disease severity.

## Methods

### Patient sample and clinical data collection

We conducted a cross-sectional study of general pediatric inpatients who were screened for respiratory viruses at a public Singaporean children's hospital, from December 2016 to April 2017. Inclusion criteria for the study were simply hospitalized patients who received a screening viral respiratory panel. This panel was ordered by the attending medical team in charge of the patients' care and was independent of the study team. The screening panel was ordered within 24 h of respiratory symptom onset and may have been ordered in conjunction with other microbiological testing depending on the patient case. Upon receiving informed consent from these patients and the parents or guardians of these patients, residual samples from clinical respiratory specimens were obtained from the in-house clinical microbiology laboratory (CML) for processing. Patient clinical data, including presenting symptoms and clinical interventions, were collected by chart review after discharge. We selected the pediatric population as this demographic is commonly afflicted with respiratory infections. This study was approved by the SingHealth IRB (CIRB Ref No.: 2016/2952) and the Duke IRB (Ref No.: Pro00079130).

### Sample processing

The CML screens clinical respiratory samples with the D3 Double Duet DFA (Direct Fluorescent Antibody) Respiratory Virus Screening and ID Kit (REF: I-01-320000, Quidel, San Diego, CA, USA) for influenza virus types A and B, respiratory syncytial virus (RSV), metapneumovirus (MPV), adenovirus, and parainfluenza virus types 1, 2, and 3. Samples positive for influenza A, B or adenovirus were sent to the Ministry of Health, Singapore laboratory for further processing and were not included in our study.

DNA was extracted from the residual respiratory samples using Qiagen DNA Blood Mini Kit (Cat No 51106: Qiagen, Hilden, Germany), following the instructions provided by the manufacturer. Previously identified primers, covering the small T antigen of KI and the VP2 regulatory region of WU, were used for TaqMan^TM^ real-time PCR (qPCR) (16). Specific primers and probes included:

KI forward (KI-B-4603-F): GAATGCATTGGCATTCGTGA
KI reverse (KI-B-4668-R): GCTGCAATAAGTTTAGATTAGTTGGTGC
KI probe (KI-B-4632-TM): fam-TGTAGCCATGAATGCATACATCCCACTGC-tamra
WU forward (WU-C-4824-F): GGCACGGCGCCAACT
WU reverse (WU-C-4898-R): CCTGTTGTAGGCCTTACTTACCTGTA
WU probe (WU-C-4861-TM): fam-TGCCATACCAACACAGCTGCTGAGC-tamra

All respiratory samples were initially screened using qPCR conducted on a BIO-RAD CFX 96 (Hercules, CA, USA) with the QIAGEN QuantiNova Probe PCR kit (Cat No: 208254, Qiagen, Hilden, Germany). Briefly, the mix contained 10 μL of probe PCR Master mix, 0.8 μL each of 10 μM forward and reverse primer, 0.4 μL of 10 μM probe, 3 μL of nuclease-free water, and 5 μl of sample nucleic acid extract in a final reaction volume of 20 μL. The samples were cycled under the following conditions: initial denaturation of 15 min at 95°C, 55 cycles of denaturation at 95°C for 15 s, and extension at 60°C for 1 min. Included in each experiment was a negative (no template) control and a positive control of a oligonucleotide taken from the VP2 region of the WU strain S3 (Genbank number EF444552) and the small T antigen region of the KI Stockholm 380 strain (Genbank number EF127908) (17). Positive samples were tested again in duplicate under the same conditions and the Ct values were averaged (Supplementary Table 1) and were subsequently sequenced for confirmation using previously published sequencing PCR methods (1).

### Statistical methods

Statistical analyses were performed using Stata 14 (Stata Corp, College Station, TX, USA). Initial bivariate screening was conducted between selected covariates of interest and various outcome variables representing respiratory illness severity. A student's *t*-test for continuous variables, and chi-square or Fisher exact tests for dichotomous or categorical variables, were used. Predictors with a bivariate *p* < 0.1 were then included in a stepwise, backward elimination unconditional generalized linear regression model. Predictors with a *p* < 0.05 were retained in the final model and adjusted prevalence ratios calculated. Outcome variables that were assessed included severe acute respiratory illness (SARI) as defined by the World Health Organization (fever >38C, cough, onset within last 10 days, and requires hospitalization), oxygen delivery, intravenous fluids, length of stay greater than or equal to 3 days, receiving oral antibiotics during admission, and prescription of a bronchodilator (18).

## Results

A total of 214 patients and parents or guardians of patients granted informed consent and were enrolled into the study. Data from nine subjects were removed due to insufficient quantity of residual nasopharyngeal samples. Four more patients were also removed as their paper charts were not traceable. In total, 201 residual samples from unique patients were collected (200 nasopharyngeal swabs and one endotracheal lavage from an intubated patient). Subjects were mostly male (56%) with a mean age of 45 months (Table 1). Seventy percent (141/201) of patients enrolled met criteria for SARI.

**Table 1:** Characteristics of 201 pediatric study subjects and an examination of their risk factors for severe acute respiratory infection (SARI), Singapore December 2016 to April 2017.

	**Patients without SARI *n* = 60**	**Patients with SARI *n* = 141**	**Unadjusted comparisons**	**Adjusted comparisons**
Mean age (months)	47.4	44.2	*p*-value = 0.69	
Gender				
Male Female	35 (58%) 25 (42%)	79 (56%) 62 (44%)	–	–
Age-group				
Less than 32 months 32 months or older	24 (40.00%) 36 (60.00%)	74 (52.48%) 67 (47.52%)	–	–
Presence of Runny Nose on Admission				
Yes No	23 (38.33%) 37 (61.67%)	85 (60.28%) 56 (39.72%)	PR = 1.31 95% CI (1.08–1.59)	PR = 1.27 95% CI (1.06–1.51)
Asthma diagnosis				
Yes No	9 (15%) 51 (85%)	6 (4%) 135 (96%)	PR = 0.55 95% CI (0.29–1.03)	-Not Significant-
Pneumonia diagnosis				
Yes No	1 (1.67%) 59 (98.33%)	25 (17.73%) 116 (82.27%)	PR = 1.45 95% CI (1.27–1.65)	-Not Significant-
Received oral antibiotics during admission				
Yes No	14 (23%) 46 (77%)	74 (52%) 67 (48%)	PR = 1.42 95% CI (1.19–1.69)	PR = 1.39 95% CI (1.17–1.65)
Molecular detection of WU				
Yes No	4 (6.7%) 56 (93.3%)	22 (15.6%) 119 (84.4%)	PR = 1.24 95% CI (1.03-1.51)	-Not significant-
Molecular detection of KI				
Yes No	0 (0%) 60 (100%)	6 (3.16%) 135 (95.74%)	–	–
Immunofluorescent detection of adenovirus				
Yes No	0 (0%) 60 (100%)	2 (1.42%) 139 (98.58%)	–	–
Immunofluorescent detection of parainfluenza virus				
Yes No	0 (0%) 60 (100%)	4 (2.84%) 137 (97.16%)	–	–
Immunofluorescent detection of enterovirus				
Yes No	2 (3.33%) 58 (96.67%)	1 (0.71%) 140 (99.29%)	PR = 0.47 95% CI (0.09–2.34)	–
Immunofluorescent detection of RSV				
Yes No	0 (0%) 60 (100%)	8 (5.67%) 133 (94.33%)	–	–
Immunofluorescent detection of metapneumovirus				
Yes No	1 (1.67%) 59 (98.33%)	3 (2.13%) 138 (97.87%)	PR = 1.07 95% CI (0.60–1.90)	–

*Comparison of means was performed using student's t-test. Bivariate analyses were performed using the chi-square or Fisher exact test. Adjusted prevalence ratios were calculated using a stepwise, backwards elimination unconditional logistic regression model*.

Overall, WU and KI viruses were detected in 13% (26/201) and 3% (6/201) of patients, respectively. Other respiratory viruses were detected, including adenovirus, parainfluenza, enterovirus, RSV, and metapneumovirus at much lower prevalence. One percent (2/201) of patients had evidence of both WU and KI infection. There were four cases where evidence of WU virus was found with one other virus in the screening panel (parainfluenza virus, RSV, metapneumovirus, rotavirus) yet this number was too low to perform statistical analysis. There were no detected cases of polyomavirus and bacterial coinfection. WU was the most prevalent out of all the viruses detected. Fifty-eight percent of patients infected with WU met clinical criteria for SARI. The prevalence of KI in this study was too low to perform multivariate modeling. Bivariate screening of risk factors for association with SARI revealed six variables to be potentially significant predictors (*p* ≤ 0.1): presence of runny nose, diagnosis of asthma, diagnosis of pneumonia, molecular detection of WU, and receiving oral antibiotics during admission. A stepwise, backward elimination logistic regression model generated from these variables revealed that SARI patients were at higher risk of presenting with a runny nose (adjusted PR = 1.27, 95% C.I. 1.06–1.51) and receiving oral antibiotics during admission (adjusted PR = 1.39, C.I. 1.17–1.65). Neither molecular evidence of WU nor KI were significantly associated with other factors of severe respiratory disease gathered in this study (Table 1).

In an examination of risk factors for “requiring supplemental O2,” the multivariate model revealed the use of bronchodilators and evidence of RSV infection to be significant predictors (Table 2). Multivariate modeling of the outcome “use of intravenous (IV) fluids” revealed a negative association with a diagnosis of bronchitis (Table 3). The use of IV fluids and receiving oral antibiotics during admission showed a positive association with the outcome “increased length of stay (LOS),” while both diagnosis of bronchitis and asthma showed an inverse association (Table 4). Multivariate modeling to predict risk factors for oral antibiotics during hospitalization could not be completed due to lack of convergence, although a diagnosis of asthma or a diagnosis of pneumonia were trending toward significance (Table 5). When assessing prescription of bronchodilators during hospitalization as the outcome, multivariate modeling revealed a diagnosis of bronchitis and use of supplemental oxygen to have a positive association and presence of RSV to have a small positive association, while diagnosis of asthma could not be included due to failure to converge in the final model. (Table 6).

**Table 2:** Characteristics of 201 pediatric study subjects and an examination of their risk factors for the use of additional oxygen (O2), Singapore December 2016 to April 2017.

	**Patients not requiring O2 (*n* = 157)**	**Patients requiring O2 (*n* = 44)**	**Unadjusted comparisons**	**Adjusted comparisons**
Age-group				
Less than 32 months 32 months or older	77 (49.04%) 80 (50.96%)	21 (47.73%) 23 (52.27%)	—	
URTI diagnosis				
Yes No	45 (28.66%) 112 (71.34%)	2 (4.55%) 42 (95.45%)	PR = 0.16 95% CI (0.04–0.62)	-Not significant-
Asthma diagnosis				
Yes No	5 (3.18%) 152 (96.82%)	10 (22.73%) 34 (77.27%)	PR = 3.65 95% CI (2.28–5.83)	-Not significant-
Bronchitis diagnosis				
Yes No	24 (15.3%) 133 (84.7%)	15 (34.1%) 29 (65.9%)	PR = 2.14 95% CI (1.28–3.60)	-Not significant-
Use of bronchodilators				
Yes No	34 (21.66%) 123 (78.34%)	39 (88.64%) 5 (11.36%)	PR = 13.67 95% CI (5.64–33.16)	PR = 12.60 95% CI (5.17–30.68)
Diagnosis of Pneumonia				
Yes No	19 (21.66%) 138 (78.34%)	7 (88.64%) 37 (11.36%)	PR = 1.27 95% CI (0.64–2.55)	–
Molecular detection of WU				
Yes No	21 (13.38%) 56 (93.3%)	5 (11.36%) 119 (84.4%)	PR = 0.86 95% CI (0.37–1.99)	–
Molecular detection of KI				
Yes No	6 (3.82%) 151 (96.18%)	0 (0%) 44 (100%)	–	–
Immunofluorescent detection of adenovirus				
Yes No	2 (1.27%) 155 (98.73%)	0 (0%) 44 (100%)	–	–
Immunofluorescent detection of parainfluenza				
Yes No	4 (2.55%) 153 (97.45%)	0 (0%) 44 (100%)	–	–
Immunofluorescent detection of enterovirus				
Yes No	3 (1.91%) 154 (98.09%)	0 (0%) 44 (100%)	–	–
Immunofluorescent detection of RSV				
Yes No	1 (0.64%) 156 (99.36%)	7 (15.91%) 37 (84.09%)	PR = 4.56 95%CI (3.09–6.74)	PR = 1.77 95% CI (1.23–2.58)
Immunofluorescent detection of metapneumovirus				–
Yes No	3 (1.91%) 154 (98.09%)	1 (2.27%) 43 (97.73%)	–	

*Comparison of means was performed using student's t-test. Bivariate analyses were performed using the chi-square or Fisher exact test. Adjusted prevalence ratios were calculated using a stepwise, backwards elimination unconditional logistic regression model*.

**Table 3:** Characteristics of 201 pediatric study subjects and an examination of their risk factors for the use of intravenous fluids (IVF), Singapore December 2016 to April 2017.

	**Patients not requiring IVF (*n* = 139)**	**Patients requiring IVF (*n* = 62)**	**Unadjusted comparisons**	**Adjusted comparisons**
Age-group				
Less than 32 months 32 months or older	64 (46.04%) 75 (53.96%)	34 (54.84%) 28 (45.16%)	—	
Bronchitis diagnosis				
Yes No	33 (23.74%) 106 (76.26%)	6 (9.68%) 56 (90.32%)	PR = 0.45; 95% CI (0.21-0.96)	PR = 0.45; 95% CI (0.21-0.96)
Pneumonia diagnosis				
Yes No	14 (10.07%) 125 (89.93%)	12 (19.35%) 50 (80.65%)	PR = 1.62 95% CI (1.00-2.60)	-Not significant-
Molecular detection of WU				
Yes No	17 (12.23%) 122 (87.77%)	9 (14.52%) 53 (85.48%)	PR = 1.14 95% CI (0.64-2.03)	—
Molecular detection of KI				—
Yes No	4 (2.88%) 135 (97.12%)	2 (3.23%) 60 (96.77%)	PR = 1.08 95% CI (0.34-3.42)	
Immunofluorescent detection of adenovirus				
Yes No	1 (0.72%) 138 (99.28%)	1 (1.61%) 61 (98.39%)	—	—
Immunofluorescent detection of parainfluenza virus				
Yes No	3 (2.16%) 136 (97.84%)	1 (1.61%) 61 (98.39%)	—	—
Immunofluorescent detection of enterovirus				
Yes No	2 (1.44%) 137 (98.56%)	1 (1.61%) 61 (98.39%)	—	—
Immunofluorescent detection of RSV				
Yes No	5 (3.60%) 134 (96.40%)	3 (4.84%) 59 (95.16%)	—	—
Immunofluorescent detection of metapneumovirus				
Yes No	3 (2.16%) 136 (97.84%)	1 (1.61%) 61 (98.39%)	—	—

*Comparison of means was performed using student's t-test. Bivariate analyses were performed using the chi-square or Fisher exact test. Adjusted prevalence ratios were calculated using a stepwise, backwards elimination unconditional logistic regression model*.

**Table 4:** Characteristics of 201 pediatric study subjects and an examination of their risk factors in association with length of stay (LOS), Singapore December 2016 to April 2017.

	**Patients with LOS < 3days (*n* = 98)**	**Patients with LOS ≥ 3days (*n* = 103)**	**Unadjusted comparisons**	**Adjusted comparisons**
Age-group				
Less than 32 months 32 months or older	44 (44.90%) 54 (55.10%)	56 (54.37%) 47 (45.63%)		
Bronchitis diagnosis				
Yes No	26 (26.53%) 72 (73.47%)	13 (12.62%) 90 (87.38%)	PR = 0.6 95%CI (0.38–0.95)	-Not significant-
Asthma diagnosis				
Yes No	12 (12.24%) 86 (87.76%)	3 (2.91%) 100 (97.09%)	PR = 0.37 95%CI (0.13–1.03)	-Not significant-
Received IV fluids during admission				
Yes No	18 (18.37%) 80 (81.63%)	44 (42.72%) 59 (57.28%)	PR = 1.67 95%CI (1.30–2.14)	PR = 1.51 95%CI (1.18–1.93)
Received bronchodilators during admission				
Yes No	43 (43.88%) 55 (56.12%)	30 (29.13%) 73 (70.87%)	PR = 0.72 95%CI (0.53–0.96)	-Not significant-
Received oral antibiotics during admission				
Yes No	31 (31.63%) 67 (68.37%)	57 (55.34%) 46 (44.66%)	PR = 1.59 95%CI (1.21–2.08)	PR = 1.43 95%CI (1.10–1.85)
Molecular detection of WU				
Yes No	15 (17.05%) 73 (82.95%)	11 (9.73%) 102 (90.27%)		
Molecular detection of KI				
Yes No	4 (4.08%) 94 (95.92%)	2 (1.94%) 101 (98.06%)		
Immunofluorescent detection of adenovirus				
Yes No	0 (0%) 100 (100%)	2 (1.94%) 101 (98.06%)		
Immunofluorescent detection of parainfluenza virus				
Yes No	3 (3.06%) 95 (96.94%)	1 (0.97%) 100 (99.03%)		
Immunofluorescent detection of enterovirus				
Yes No	1 (1.02%) 97 (98.98%)	2 (1.94%) 101 (98.06%)		
Immunofluorescent detection of RSV				
Yes No	5 (5.10%) 93 (94.90%)	3 (2.91%) 98 (97.09%)		
Immunofluorescent detection of metapneumovirus				
Yes No	2 (2.04%) 96 (97.96%)	2 (1.94%) 101 (98.06%)		

*Comparison of means was performed using student's t-test. Bivariate analyses were performed using the chi-square or Fisher exact test. Adjusted prevalence ratios were calculated using a stepwise, backwards elimination unconditional logistic regression model*.

**Table 5:** Characteristics of 201 pediatric study subjects and an examination of their risk factors for the use of oral antibiotics (abx), Singapore December 2016 to April 2017.

	**Patients not prescribed Oral Abx (*n* = 113)**	**Patients prescribed Oral Abx (*n* = 88)**	**Unadjusted comparisons**	**Adjusted comparisons**
Age-group			–	
Less than 32 months 32 months or older	50 (44.25%) 63 (55.75%)	48 (54.55%) 40 (45.45%)		
URTI diagnosis				–
Yes No	32 (28.32%) 81 (71.68%)	15 (17.05%) 73 (82.95%)	PR = 0.67 95%CI (0.43–1.06)	
Asthma diagnosis				
Yes No	14 (12.39%) 99 (87.61%)	1 (1.14%) 87 (98.86%)	PR = 0.14 95%CI (0.02–0.95)	-No convergence-^*^-NoN
Pneumonia diagnosis				
Yes No	0 (0%) 113 (100%)	26 (29.55%) 62 (70.45%)	PR = 4.57^*^ 95%CI (1.75–7.38)	-No convergence-^*^-N
Molecular detection of WU			–	–
Yes No	11 (9.73%) 102 (90.27%)	15 (17.05%) 73 (82.95%)		
Molecular detection of KI			–	–
Yes No	4 (3.54%) 109 (96.46%)	2 (2.27%) 86 (97.73%)		
Immunofluorescent detection of adenovirus				–
Yes No	0 (0%) 113 (100%)	2 (2.27%) 86 (97.73%)	-No Convergence-^*^	
Immunofluorescent detection of parainfluenza				
Yes No	3 (2.65%) 110 (97.35%)	1 (1.14%) 87 (98.84%)	–	–
Immunofluorescent detection of enterovirus			–	–
Yes No	2 (1.77%) 111 (98.23%)	1 (1.14%) 87 (98.84%)		
Immunofluorescent detection of RSV			–	–
Yes No	4 (3.54%) 109 (96.46%)	4 (4.55%) 84 (95.45%)		
Immunofluorescent detection of metapneumovirus				–
Yes No	0 (0%) 113 (100%)	4 (4.55%) 84 (95.45%)	No Convergence-^*^-	

Comparison of means was performed using student's t-test. Bivariate analyses were performed using the chi-square or Fisher exact test.

(*) *Values for pneumonia diagnosis, detection of adenovirus, and detection of metapneumovirus were not suitable for relative risk calculation and required logistic modeling. No adjusted modeling was performed due to lack of convergence*.

**Table 6:** Characteristics of 201 pediatric study subjects and an examination of their risk factors for the use of bronchodilators during admission, Singapore December 2016 to April 2017.

	**Patients not prescribed dilators (*n* = 128)**	**Patients prescribed dilators (*n* = 73)**	**Unadjusted comparisons**	**Adjusted comparisons**
Age-group				
Less than 32 months 32 months or older	61 (47.66%) 67 (52.34%)	37 (50.68%) 36 (49.32%)	PR = 0.92 95%CI (0.64–1.34)	
Cough				
Yes No	106 (82.81%) 22 (17.19%)	71 (97.26%) 2 (2.74%)	PR = 4.81 95%CI (1.26–18.37)	-No significance-
URTI diagnosis				
Yes No	42 (32.81%) 86 (67.19%)	5 (6.85%) 68 (93.15%)	PR = 0.24 95%CI (0.10–0.56)	-No significance-
Asthma diagnosis				
Yes No	0 (0%) 128 (100%)	15 (20.55%) 58 (79.45%)	PR = 4.22^*^ 95%CI (1.39–7.05)	-No convergence-
Bronchitis diagnosis				
Yes No	9 (7.03%) 119 (92.97%)	30 (41.10%) 43 (58.90%)	PR = 2.89 95%CI (2.13–3.95)	PR = 1.82 95%CI (1.49–2.24)
Use of O2 during admission				
Yes No	5 (3.91%) 123 (96.09%)	39 (53.42%) 34 (46.58%)	PR = 4.09 95%CI (2.98–5.61)	PR = 3.67 95%CI (2.69–5.05)
Molecular detection of WU				–
Yes No	15 (11.72%) 113 (88.28%)	11 (15.07%) 62 (84.93%)	PR = 1.19; 95% CI (0.73–1.95)	
Molecular detection of KI				–
Yes No	4 (3.13%) 124 (96.87%)	2 (2.74%) 71 (97.26%)	PR = 0.92 95%CI (0.29–2.88)	
Immunofluorescent detection of Adenovirus			–	–
Yes No	0 (0%) 128 (100%)	2 (2.74%) 71 (97.26%)		
Immunofluorescent detection of Parainfluenza			–	–
Yes No	3 (2.34%) 125 (97.66%)	1 (1.37%) 72 (98.63%)		
Immunofluorescent detection of enterovirus			–	–
Yes No	3 (2.34%) 125 (97.66%)	0 (0%) 73 (100%)		
Immunofluorescent detection of RSV				
Yes No	1 (0.78%) 127 (99.22%)	7 (9.59%) 66 (90.41%)	PR = 2.56 95%CI (1.84–3.55)	PR = 1.002 95%CI (1.0019–1.002)
Immunofluorescent detection of Metapneumovirus			–	–
Yes No	2 (1.56%) 126 (98.46%)	2 (2.74%) 71 (97.26%)		

Comparison of means was performed using student's t-test. Bivariate analyses were performed using the chi-square or Fisher exact test. Adjusted prevalence ratios were calculated using a stepwise, backwards elimination unconditional logistic regression model.

(*) *Values for asthma diagnosis were not suitable for relative risk calculation and required logistic modeling. This variable was not included in adjusted comparisons as convergence could not be achieved*.

## Discussion

The prevalence ofWU and KI detected in our study was 13% (26/201) and 3% (6/201) respectively with 1% (2/201) coinfection. These data represent the first reporting, to our knowledge, of respiratory polyomaviruses in a Singaporean population. In comparison with other Asian countries, WU prevalence has been reported as 7.0% in Korea, 4.2% in China, 6.29% in Thailand, 5.3% in the Philippines, and 16.4% in Japan (3, 4, 7, 17, 19). The prevalence of KI has been reported as 1.0% in Korea, 2.7% in China, 1.99% in Thailand, 4.2% in the Philippines, and 3.0% in Japan (3, 4, 7, 17, 19). The prevalence of WU in our study was higher than many other Southeast Asian countries and may be attributable to our use of a more sensitive qPCR detection methods as used in Teramoto et al. in Japan (19). This study, like many previous prevalence studies, was not able to incorporate asymptomatic controls, however WU has been detected at proportions of 4.2 and 6.3% in children without respiratory symptoms (3, 20). Additional patients with influenza and adenovirus infections were not available for study which could alter our study population to detect a higher prevalence of WU or KI.

Although previous studies suggest respiratory polyomaviruses as a cause of respiratory disease, pathogenicity has not been proven (2, 8–13, 21). If polyomaviruses were a clinically significant contributor to severe respiratory disease in the pediatric population one might expect infected individuals to have more severe presenting symptoms, more severe diagnoses, and require more interventions. Although presence of WU virus trended toward a significant association with SARI, it was not associated with any of the interventions or outcomes we studied. We interpret the lack of significant differences in clinical diagnoses or severe disease interventions between polyomavirus-positive and polyomavirus-negative patients to suggest that the overall clinical burden of WU, at least in this small study, was small. In contrast, RSV was associated with the use of supplemental oxygen (Table 2) which demonstrated RSV's recognized increase in respiratory morbidity.

This study continues the trend toward detailed clinical reporting surrounding polyomavirus infections to produce more insightful knowledge regarding the association between these viruses and human disease as started by several groups (7, 17, 22, 23). In their study, Rao et al. studied a much larger pediatric cohort and suggested a pathogenic role for KI virus based on the association between viral load and reported disease severity in Filipino patients with lower respiratory tract disease, but reported no such association with WU (7). While the low number of KI positive patients in our study precludes the ability to comment on pathogenicity, the fact that all six KI patients met clinical criteria for SARI and were negative for non-polyomaviruses suggests that more clinical data should be gathered on KI positive patients. Both Rao et al. and our study are only cross-sectional snapshots of acute hospitalizations. A longitudinal study would be more valuable as it could permit a closer examination of morbidity, such as a reactivation or exacerbation of the symptoms of asthma (24–26).

Other study limitations include a lack of clinically established cutoffs for determining respiratory polyomavirus infections based on molecular detection mechanisms. This is a consistent problem across studies such as ours, as the clinical impact of respiratory polyomaviruses has not been fully established. Given that the association between molecular evidence of WU and SARI was trending toward significance, our study might have benefitted from increasing the sample size to further elucidate any possible association. Inherent in a study addressing clinical significance, there might be other causative pathogens in symptomatic patients which were not detected or were failed to be detected given the screening DFA clinical assay. Our study had a low viral co-infection prevalence and thus we cannot comment on how respiratory polyomavirus infection affects children already infected with another virus. Although most of our samples came from nasopharyngeal swabs, there is inherent variability in sampling techniques. All patients were screened with a respiratory virus diagnostic panel that did not include rhinovirus or bacterial pathogens. Bacterial pathogen diagnostics ordering was dependent on the clinical team and not often requested. In addition, the use of antibiotics varied based on the medical team and the case, with only some teams discontinuing antibiotic therapy after a positive viral screen. Finally, our study would have been strengthened through the additional study of asymptomatic controls to provide a reference point and we would want to include this population in future studies. However, the premise of this pilot study was to establish the prevalence of respiratory polyomaviruses in pediatric patients with respiratory symptoms in Singapore, which was found to be higher than other Southeast Asian countries, and to explore the clinical burden of these viruses for which further studies are indicated.

## Conclusions

We determined the prevalence of WU to be 13% and KI to be 3% which is high compared to other pediatric populations in SE Asia. Our results do not suggest that the molecular presence of WU in this Singaporean pediatric population with respiratory symptoms was associated with SARI or clinical interventions suggestive of severe respiratory illness.

## Author contributions

CH-E, GG, KT, and JL contributed toward conception and design of the study. CH-E and KT recruited patients and collected samples. CH-E performed the chart review, organized the database, and performed the molecular assays. CH-E, BA, and KC performed the statistical analysis; CH-E, KC, and GG wrote the first draft of the manuscript. All authors contributed to manuscript revision, read and approved the submitted version.

### Conflict of interest statement

The authors declare that the research was conducted in the absence of any commercial or financial relationships that could be construed as a potential conflict of interest.
