# Supplementary material for: Prevalence of Respiratory Polyomaviruses Among Pediatric Patients With Respiratory Symptoms in Singapore
Source: Front Pediatr. 2018 Aug 17;6:228. doi: 10.3389/fped.2018.00228 (PMC6107759; doi:10.3389/fped.2018.00228)
Supplement: Supplementary file 1 [file Table_1.DOCX]

Supplementary Material

Prevalence of Respiratory Polyomaviruses among Symptomatic Pediatric Patients in Singapore

Christophe Hansen-Estruch^1,2^*, Kristen K. Coleman PhD^1^, Koh C. Thoon MD^3^, Jenny G Low MD^1,4^, **Correspondence:** ch294@duke.edu

# Supplementary Data

Supplemental Table1: Results of polyomavirus quantitative PCR (qPCR) as expressed in cycle threshold (Ct) values. All respiratory samples were tested with qPCR and the Ct values of positive samples are listed in the initial test column. Every positive sample was retested with the same qPCR performed in duplicate with the average Ct values presented in the second test/average column. There were five samples that were positive for WU on the initial test but were negative on secondary testing and were not counted as positive in the final results (data not shown).

|  |  |
| --- | --- |
